# Synapse Loss and Dendrite Remodeling in a Mouse Model of Glaucoma

**DOI:** 10.1371/journal.pone.0144341

**Published:** 2015-12-04

**Authors:** Ryan H. Berry, Juan Qu, Simon W. M. John, Gareth R. Howell, Tatjana C. Jakobs

**Affiliations:** 1 Harvard Medical School, Department of Ophthalmology, Massachusetts Eye and Ear Infirmary, Boston, MA 02114, United States of America; 2 The Jackson Laboratory, 600 Main Street, Bar Harbor, ME, United States of America; 3 The Howard Hughes Medical Institute, The Jackson Laboratory, 600 Main Street, Bar Harbor, ME, United States of America; 4 Department of Ophthalmology, Tufts University School of Medicine, Boston, MA, United States of America; University of Nebraska Medical Center, UNITED STATES

## Abstract

It has been hypothesized that synaptic pruning precedes retinal ganglion cell degeneration in glaucoma, causing early dysfunction to retinal ganglion cells. To begin to assess this, we studied the excitatory synaptic inputs to individual ganglion cells in normal mouse retinas and in retinas with ganglion cell degeneration from glaucoma (DBA/2J), or following an optic nerve crush. Excitatory synapses were labeled by AAV2-mediated transfection of ganglion cells with PSD-95-GFP. After both insults the linear density of synaptic inputs to ganglion cells decreased. In parallel, the dendritic arbors lost complexity. We did not observe any cells that had lost dendritic synaptic input while preserving a normal or near-normal morphology. Within the temporal limits of these observations, dendritic remodeling and synapse pruning thus appear to occur near-simultaneously.

## Introduction

Glaucoma is a progressive neurodegenerative disease which ultimately leads to the loss of retinal ganglion cells [1]. The death of the ganglion cells occurs by apoptosis [2] and is preceded by a remodeling of the dendritic arbor, shrinking of the soma, and axonal atrophy [3]. Recent experimental evidence indicates that the overt remodeling of the dendritic arbors may be preceded by more subtle functional impairment, possibly caused by a loss of synapses on the dendrites of the ganglion cells [4].

The elimination of synapses from neuronal circuits is an important phenomenon both in developmental maturation and in pathological conditions. In development, initially an abundance of synapses is formed, many of which are lost as the circuit matures, whereas the remaining synapses are strengthened. Microglial cells, the resident immune cells of the CNS, play a key role in eliminating supernumerary synapses [5, 6]. Microglia have been shown to engulf and phagocytose synaptic material [7]. Recently, the complement system has been found to be involved in this process. In a manner quite similar to their function in immunity, complement factors opsonize synaptic structures and tag them for elimination by the microglia [7–9]. In glaucoma, complement factors, including C1q and C3 are upregulated both at the mRNA and the protein level in both the retina and optic nerve head [8, 10, 11]. Furthermore, indicating an important role for the complement cascade in glaucoma, deletion of the complement component *C1qa*, protected ganglion cells from degeneration in a mouse model of glaucoma [11]. However, the role of complement in glaucoma is not clear. One possible role is that, similar to remodeling of RGC synapses in development [7, 8], the re-expression of complement factors in the retina leads to the elimination of synapses on retinal neurons, especially on the ganglion cells, causing early dysfunction of RGCs [8, 12].

At present, there is no direct morphological evidence for the involvement of microglia in synapse elimination in the retina. An additional problem with this model is also that the synapses in retinal ganglion cells do not reside on spines but are located directly on the dendritic shaft [13], which would make direct engulfment and phagocytosis of the synapse by microglia difficult. PSD-95 expression and synaptic localization can be controlled by the neuron itself. As shown by time-lapse studies, PSD-95-GFP undergoes dynamic movement to and from synaptic sites [14]. Mechanisms of this turnover include for example palmitoylation and depalmitoylation [15] or ubiquitination of PSD-95 [16], none of which would require the direct involvement of microglia.

To assess the question whether synapse elimination precedes remodeling of ganglion cell dendrites, we observed the frequency and distribution of excitatory synapses on retinal ganglion cells in a mouse model of glaucoma. For this purpose, we transfected retinal ganglion cells in vivo in the retinas of 11 months old DBA/2J mice. DBA/2J mice develop increased intraocular pressure and glaucomatous loss of ganglion cells as a result of an iris stroma disease by a mechanism that is similar to pigmentary glaucoma observed in humans [17, 18]. Importantly, the degeneration of ganglion cells in DBA/2J retinas is asynchronous, so that within the same eye ganglion cells that are quite normal or in early stages of remodeling coexist with frankly degenerated cells that have lost most of their dendrites [3, 19, 20].

We labeled excitatory synapses on ganglion cell by an AAV-mediated expression of GFP-tagged PSD-95, the major structural component of glutamatergic synapses [21]. The expression of PSD-95-GFP fusion proteins lends itself to the in vivo labeling of excitatory synapses because PSD-95 is abundant enough in the postsynaptic density to allow for the light microscopic visualization of individual synapses and the fusion of GFP to PSD-95 does not seem to interfere with the transport and synaptic localization of the protein. Consequently, expression of PSD-95-GFP has been used to identify post-synaptic densities in a variety of neurons, both in cell culture and in living animals [4, 22–29].

We also used optic nerve crush as a model of faster degeneration of ganglion cells. In both models, the number and density of synapses on ganglion cell dendrites decreases significantly and the cells’ dendrites show morphological signs of remodeling. We did not observe instances of ganglion cell dendrites that were devoid of synaptic input without being obviously remodeled.

## Materials and Methods

### Animal housing

C57BL/6J, DBA/2J, and DBA/2J-*Gpnmb* ^+^ mice were obtained from The Jackson Laboratory (Bar Harbor, ME). All animal procedures were approved by the Institutional Animal Care Committees of the Massachusetts Eye and Ear Infirmary and Schepens Eye Research Institute. Anesthesia was performed using an injection of ketamine/xylazine. Animal euthanasia was performed by CO_2_ asphyxiation. DBA/2J mice served as an inherited mouse model of glaucoma [18]. Eleven months old DBA/2J mice were used, because mice frequently show moderate and severe glaucomatous damage at this age. For strain-matched controls, one year old DBA/2J-*Gpnmb* ^*+*^ mice, which develop a milder form of the iris disease compared to DBA/2J mice, but not glaucoma, were used [30]. C57BL/6J mice that received an optic nerve crush to the left eye served as an acute model RGC axon damage.

### Tissue preparation

Mice were anesthetized with an intraperitoneal injection of ketamine and xylazine, and then sacrificed by CO_2_ asphyxiation followed by cervical dislocation. The optic nerves of exposed eyes were cut with sharp scissors. Enucleated eyes were immediately fixed for 15 min in 4% paraformaldehyde to strengthen the structural integrity of the retina prior to handling. For retina extractions, eyes were hemisected along the ora serrata in a 0.1 M solution of phosphate buffer. Retinas were delicately teased off the pigment epithelium, and four curvature-relieving cuts were made before mounting the retinas ganglion cell side up on nitrocellulose filters. The retinas were then fixed again for 1 h in 4% paraformaldehyde and processed for immunohistochemistry.

### Preparation of virus and intravitreal injections

PSD-95 is a scaffolding protein that localizes to excitatory postsynaptic sites [22]. PCR amplification of the coding region of murine PSD-95 was performed on brain cDNA to derive PSD-95-GFP, which was then subcloned into an adeno-associated viral vector, producing the AAV-PSD-95-GFP virus. The virus preparation was performed by the Harvard Gene Therapy Initiative (Harvard Medical School, Boston, MA). Mice were infected with 0.5–1μL AAV-PSD-95-GFP via intravitreal injection. A glass micropipette (~100μm) was used to deliver the virus at a location slightly posterior to the limbus. All mice were incubated with the virus for two weeks prior to sacrifice.

### Optic nerve crush

The left eyes of C57BL/6 mice were subjected to an optic nerve crush after a two-week incubation period with AAV-PSD-95-GFP. Optic nerve crush was performed as described previously [31, 32]. In brief, mice were anesthetized and the conjunctiva and extraocular muscles were separated to expose the optic nerve. The optic nerve was clamped for 10 sec, approximately 100 μm behind the globe, using self-closing jeweler’s forceps. Mice were sacrificed and retinas extracted seven days following surgery. The right eyes served as controls.

### Immunohistochemistry

To visualize retinal ganglion cells (RGCs), retinas were stained with a monoclonal antibody against SMI-32 neurofilament (1:400, Covance, Dedham, MA). Antibody staining was revealed with an Alexa Fluor 598 conjugated secondary antibody (Jackson Laboratories, West Grove, PA).

### Image Acquisition

For images of whole-mount retinas, z-stacks with a step size of 1.5 μm were taken with a 10X glycerol-immersion objective (HC PL APO 10.0x0.40 IMM Leica, Germany); each stack ran through the entire depth of the retina. These z-stacks were then stitched together utilizing the automated feature included in the Leica Application Suite (Leica Microsystems, Buffalo Grove, IL) to produce maximum intensity projections of the whole retina. For images of individual neurons, two-channel z-stacks with a step size of 0.5 μm were taken with a Leica TCS SP5 confocal microscope, using a 63X glycerol-immersion objective (HCX PL APO C5 63.0x1.30 Glyc 21°C UV, Leica, Germany) at 2X optical zoom; each stack began at the soma and ended where the dendritic arbor was no longer apparent. A frame average of three was used to minimize background noise that might obscure synapse counts. The ten highest-quality cells were selected from each condition for imaging. The overall brightness and contrast of images were adjusted with Photoshop CS5 (Adobe Systems, San Jose, CA).

### Analysis of glaucoma damage

Two blinded observers assessed the degree of axon loss visualized by gaps in the SMI-32 stain in retinal whole-mounts. Retinas were classified as having either “no or early glaucoma” for those with no readily apparent axon loss, “moderate glaucoma” for those that lost many axons, or “severe glaucoma” for those where >50% axons were lost. We recently provided further support for this classification scheme in a study that showed a significant correlation between SMI-32 staining of the ganglion cell axons and the cell counts in the ganglion cell layer; use of SMI-32 also provides the added benefit of revealing whether a cell of interest originates from a degenerated or relatively normal sector [33].

### Digitization of dendrites and puncta

ImageJ (National Institute of Health, Bethesda, Maryland), specifically the “Simple Neurite Tracer” plugin, was used to trace dendritic arbors of individual ganglion cells and mark the locations of PSD-95 puncta within confocal image stacks. SMI-32 staining allowed the dendritic trees to be traced without difficulty in cells from retinas subjected to DBA/2J glaucoma or an optic nerve crush in the case of C57/BL6 mice. Since the objective’s field of view could not capture the entire dendritic arbor and dendrites from nearby neurons sometimes obscured tracing efforts, only partial reconstructions of neurons, were possible in any condition. Each trace was analyzed by another observer before it was included in the study. Once a dendritic arbor was traced, a z-stack of the trace was created to facilitate marking of PSD-95 puncta. Excitatory input was marked using the point tool. To ensure that only puncta belonging to a neuron of interest were counted, the z-stack of the traced dendrites and the z-stack of the channel containing PSD-95 labeling were merged. Brightness and contrast of the PSD-95 z-stack was adjusted to facilitate the laying of puncta. Two condition-blinded observers marked the positions of puncta for each dendritic arbor. The x, y, and z coordinates of each punctum were saved in a text file for later analysis. Images were generated by maximum intensity projections of traced z­stacks overlaid by markers for each individual puncta. The raw data files were submitted to the Harvard Dataverse and can be accessed at: http://dx.doi.org/10.7910/DVN/NPUKBY.

### Statistical analysis

Dendritic length was computed using the “Simple Neurite Tracer” plugin in ImageJ. Complexity of a cell was computed by counting the number of non-terminal nodes that appeared in the trace of a cell’s dendritic arbor. The overall linear density of puncta for a cell was computed by dividing the total number of puncta marked in a cell by the length of the cell’s entire dendritic arbor. The linear density of the densest branch was also computed by selecting the respective branches by visual inspection. The number of puncta on the branch segment of interest was then divided by the length of that branch segment. Since each cell was marked with puncta twice, an average was obtained from these markings and the resulting value was used for further calculations.

Differences between experimental groups were investigated using either a Student’s t-test or a single­factor analysis of variance (ANOVA), and use of each test is indicated when necessary.

## Results

### Linear density of excitatory input to retinal ganglion cells in *Gpnmb+* mice

**Fig 1:**
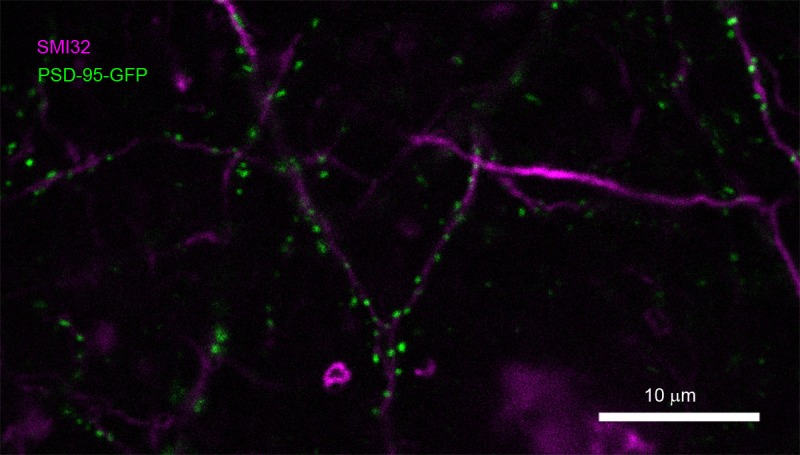
Labeling excitatory synaptic input on retinal ganglion cells. The synaptic sites were labeled with PSD-95-GFP that was delivered into retinal ganglion cells by in-vivo transfection with AAV2 (green). The dendrites were counterstained with an antibody against neurofilament (SMI32, magenta).

**Fig 2:**
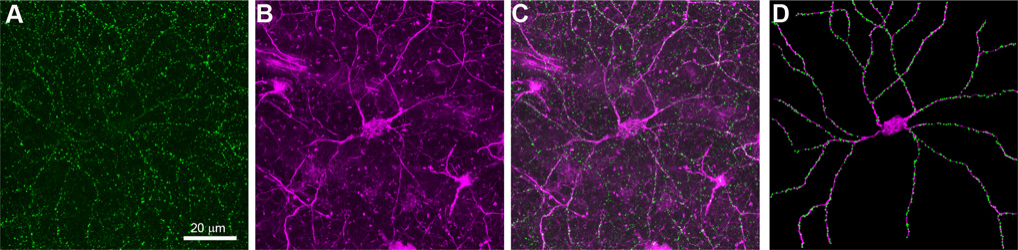
Labeling synaptic input on individual retinal ganglion cells in non-glaucomatous retinas. A, Excitatory synaptic inputs were labeled by AAV2-mediated transfection of ganglion cells with PSD-95-GFP, leading to a punctuate labeling. B, To assign synaptic puncta to individual ganglion cells, the cytoskeleton of large ganglion cells was labeled with an antibody against neurofilament. C, Overlay of A and B. D, Digital representation of the dendritic arbor with assigned synaptic input sites.

To visualize excitatory input to retinal ganglion cells in mice we used PSD-95-GFP, a well-established marker for excitatory postsynaptic densities. Expression was achieved through intravitreal injections of an adeno­associated viral (AAV, serotype 2) vector containing PSD-95-GFP administered to each eye followed by a two-week incubation period to allow the virus to infect cells in the retina. This method of labeling has two advantages over immunostaining. First, it facilitates counts of PSD-95-GFP puncta due to the relatively sparse labeling it provides. An antibody, in contrast, would visualize all excitatory synapses in the inner plexiform layer (IPL), making it difficult to unambiguously assign synapses to an individual ganglion cell. Second, the use of the viral vector ensures that transfected neurons express the marker throughout the entirety of their dendritic arbors, preventing instances of undercounting puncta due to variable antibody staining. We first assessed excitatory inputs in 11-month old DBA/2J-*Gpnmb* ^*+*^ (D2-*Gpnmb* ^+)^ control mice. Virus-transfected neurons displayed fluorescent PSD-95-GFP puncta throughout their dendritic arbors (Fig 1). To discern which PSD-95-GFP puncta belonged to RGCs, we stained each retina with an antibody against SMI32 neurofilament (Fig 2B). The antibody labels the cytoskeleton of all RGC types but exhibits a relative preference for α­ganglion cells, the largest ganglion cells found in the mouse retina. We collected confocal image stacks to examine the linear density of PSD-95-GFP puncta along dendritic segments of neurons brightly labeled by SMI32 (Fig 2C).

To better visualize the cell of interest and facilitate later quantification, we digitized the RGC contained within each image stack. Of the 20 cells that were imaged from two D2-*Gpnmb* ^*+*^ retinas, we selected the first 10 cells of suitable quality for digitization. Using ImageJ, we traced the dendritic arbor of each neuron in three dimensions following the SMI32 stain as a guide through the z-stack and irrespective of the channel containing the PSD-95-GFP. Only those branches that we were certain belonged to the cell of interest were traced, likely artificially decreasing the complexity of the digital reproductions. We then placed markers wherever PSD-95-GFP puncta lied along these traced dendritic trees (Fig 2D).

In these imaged stacks, we found, through visual observations, PSD-95-GFP puncta to be regularly distributed across the dendritic arbor of SMI32+ cells, agreeing with observations made in a number of other mammalian models [29]. Only primary branches and proximal branches that crossed levels of the IPL showed relatively fewer puncta (data not shown). From an anatomical standpoint, relatively lower densities in primary branches would be expected since these dendritic segments have not reached the stratification depth at which they receive the majority of their synaptic input [28].

### Effects of DBA/2J glaucoma on dendritic complexity and linear density

**Fig 3:**
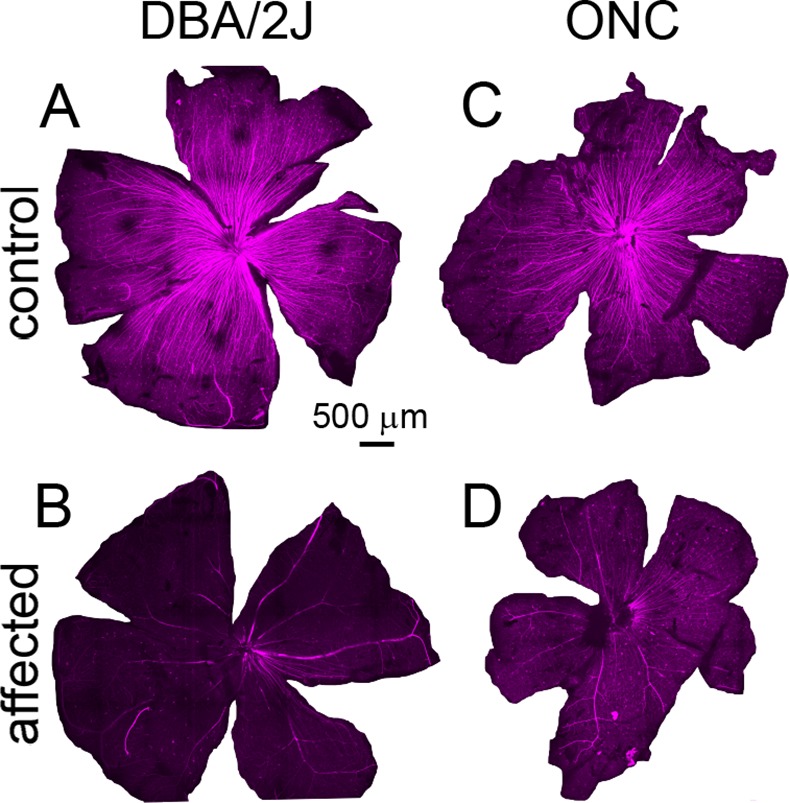
Retina whole-mounts stained for the axonal marker SMI32 (neurofilament). A, D2.Gpnmb+ mice with no axon loss. B, retina from a 11 month old DBA/2J mouse with severe loss of ganglion cells and axons. C. Control retina from a C57bl/6 mouse. D. Retina from a C57bl/6 mouse 7 days after optic nerve crush.

We repeated the same protocol for cells obtained from retinas from DBA/2J mice. For these experiments, we used eleven-month old DBA/2J mice. Since glaucoma severity is variable between mice at this age, we graded the severity of glaucomatous damage using similar guidelines to a well-established three-point scale [18, 33] (Fig 3). Only retinas that received a grading of “severe” (3 retinas) were included in the study to fully appreciate the effects of long-term glaucoma on excitatory input to RGCs. We collected image stacks of 19 cells from two such retinas and, again, selected for digitization 10 typical cells that were of suitable quality; however, many more cells were inspected microscopically.

**Fig 4:**
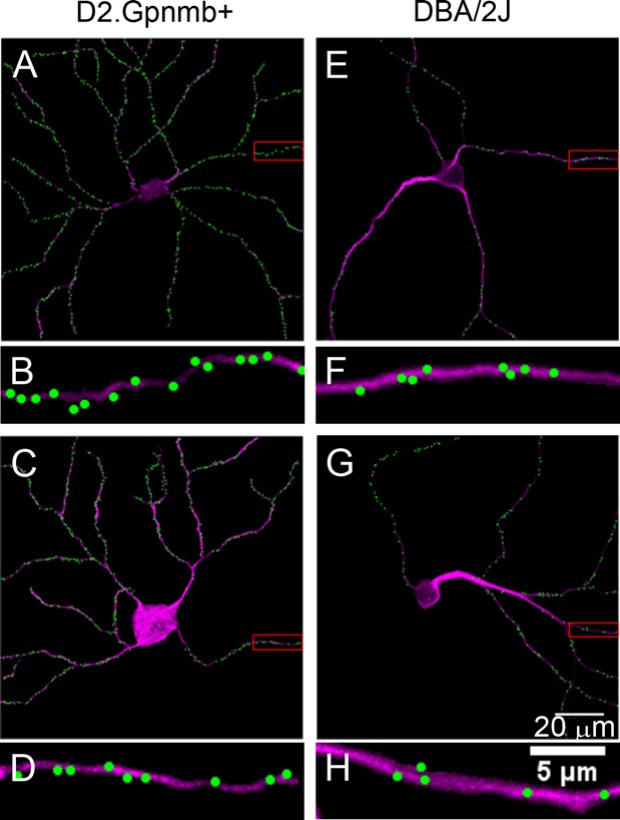
Labeling of synaptic inputs to ganglion cells in glaucomatous DBA/2J retinas. A, Digital representation of a non-glaucomatous (D2.Gpnmb+) retina. B, Higher magnification of the boxed area in panel A, showing the density of excitatory synaptic inputs to a higher-order dendrite. C & E, Two examples of ganglion cells from glaucomatous DBA/2J mice. The dendritic remodeling is obvious. D & F, Higher magnification views of the boxed areas in panels C and E, respectively. Overall synaptic density is lower than in the non-glaucomatous ganglion cells.

SMI32+ labeling in DBA/2J retinas revealed under-branched RGCs, a known characteristic of retinas subjected to glaucomatous damage [3]. In most cells, only primary, secondary, and tertiary dendrites were present (Fig 4E and 4G). To quantify the effects of DBA/2J glaucoma on dendritic complexity, we attributed a “complexity” value to each of the 10 digitized cells by counting the non­terminal nodes within each dendritic arbor. We found that cells from DBA/2J retinas experienced on average a 30% decrease in complexity when compared to controls (D2-*Gpnmb* ^*+*^: 12.8 non-terminal nodes v. DBA/2J: 9 non-terminal nodes, Table 1). This decrease likely reflects a slight underestimate, because SMI32 staining was weaker in control retinas than in DBA/2J retinas, hindering tracing efforts and, in turn, artificially decreasing complexity values for control cells; it has been noted that SMI-32 staining is stronger in cells of DBA/2J retinas, possibly due to abnormal accumulation of neurofilament in these cells [3].

**Table 1:** Mean Overall Linear Density and Complexity Values across Experimental Conditions. Mean values for overall linear density and complexity are shown with associated standard errors of the mean (n = 10 cells for each condition). Comparisons between experimental conditions and corresponding controls are indicated by significance values generated via two-tailed T-tests.

	Experimental Conditions			
	D2.Gpnmb+	DBA/2J (11 mth)	ONC control	ONC experimental
**Mean Overall Linear Density**	0.47	0.34	0.63	0.32
SEM	0.03	0.02	0.03	0.02
P-Value		1.4 E-3		2.76 E-7
**Mean Complexity Value**	12.80	9.00	14.30	9.60
SEM	1.07	1.15	0.79	0.86
P-Value		2.68 E-2		7.88 E-4

Visual inspection of linear density in the digitized DBA/2J cells gave the impression that these cells, on average, experienced losses in input when compared to D2-*Gpnmb* ^*+*^ controls. Overall linear density appeared to be lower in most cells while a few appeared to retain control levels of input. Cells that appeared to retain control levels of overall linear density often seemed to display D2-*Gpnmb* ^*+*^ levels of dendritic complexity. Cells with overall linear densities below control levels often exhibited dendritic under-branching. In these cells with fewer inputs, losses appeared throughout the cell especially in more proximal branches, which appeared thicker and comprised a larger portion of the total dendritic arbor due to the loss of higher order branches (Fig 4E and 4G).

We first quantified these changes in linear density of excitatory input by examining the overall linear density of each cell (number of marked puncta per cell/length of total dendritic arbor). We found that DBA/2J cells experienced on average a 28% decrease in linear density when compared to D2.*Gpnmb+* controls (D2-Gpnmb^+^: 0.47μm^-1^ v. DBA/2J: 0.34μm^-1^, Table 1).

**Fig 5:**
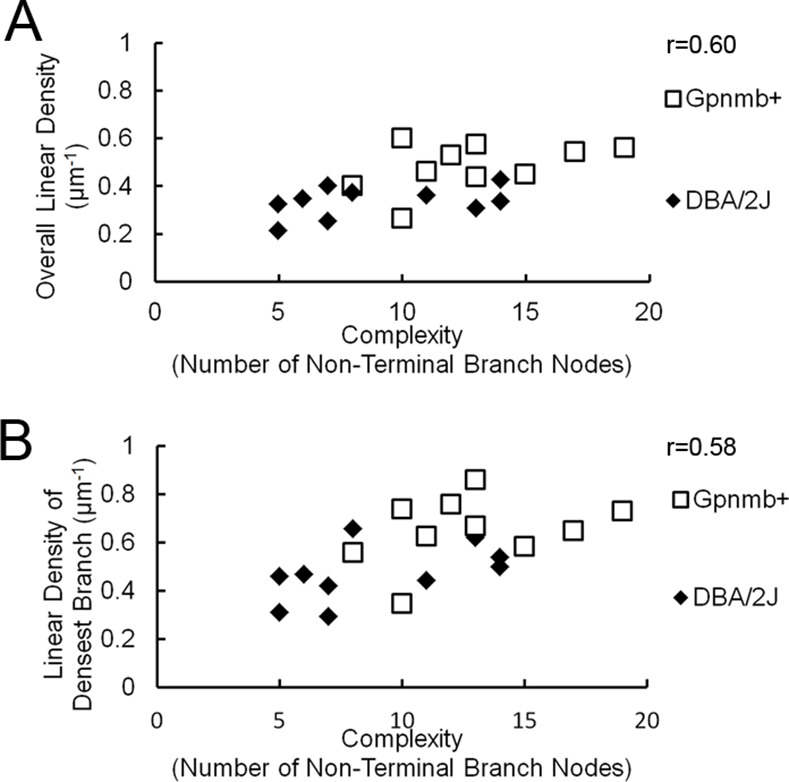
Correlation of synaptic density with complexity of the dendritic arbor in glaucomatous DBA/2J and control D2.Gpnmb+ retinas. A, Scatterplot of overall linear density of PSD-95 puncta reveals a weak correlation with the complexity of the dendritic arbor as measured by the number of non-terminal nodes. B, the correlation was repeated for the densest branch.

We then attempted to correlate changes in linear density with the complexity of the dendritic arbor for each cell. DBA/2J cells with dendritic complexity values near those of D2-*Gpnmb* ^*+*^ cells tended to also have linear density values near those of D2-*Gpnmb* ^*+*^ cells. In addition, DBA/2J cells with less complex values possessed linear densities lower than those of controls (Fig 5A). To be sure that these losses were not simply the result of primary branches comprising a greater portion of the total dendritic arbor, we also examined the relationship between complexity values and the linear density of the most dense branches visualized in the dendritic arbor. For these experiments, we plotted the most and least dense branch of each cell against the complexity of the cell’s dendritic arbor. We found that complexity weakly correlated with the value of the most dense branch segment (Fig 5B). Both dendritic complexity and linear density of PSD labeled puncta are lower in the glaucomatous DBA/2J mice, but neither seems to be primarily affected, so there is no evidence that synaptic density or dendritic complexity change at appreciatively different rates.

### Effects of optic nerve crush on dendritic complexity and linear density

Because the time of the insult is better controlled, we repeated the experimental procedure in C57BL/6J mice that received an optic nerve crush in one eye, utilizing the unaffected eye as a control. We collected 10 cells from the retina of the control eye and 22 image stacks from the retina of the crushed eye and digitized the first 10 cells that were of high enough quality.

**Fig 6:**
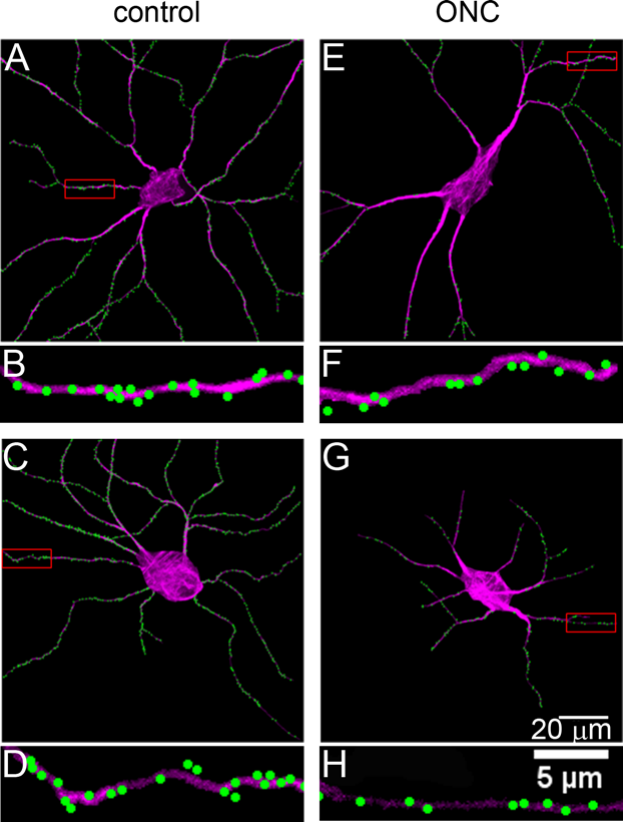
Labeling of synaptic inputs to ganglion cells after optic nerve crush. A, Normal cell from the contralateral (uncrushed) eye. B, Higher magnification of the boxed area in panel A. C & E, Two examples of ganglion cells one week after optic nerve crush. Note the decreased complexity of the dendritic arbors. D & F, Higher magnification of the boxed areas in panels C and E.

SMI32 labeling of the crushed retina revealed the effects of optic nerve crush on dendritic complexity. RGCs from the crushed eye appeared to exhibit dendritic under-branching when compared to right-eye controls (Fig 6). Most of these cells possessed thick proximal dendrites, paralleling the changes seen as a result of DBA/2J glaucoma (Fig 6E). Other cells possessed thinner dendrites that did not extend far from the soma (Fig 6G). The rest of these cells appeared unaffected, possessing dendritic complexity and thickness near that of cells from the control eye (Data Not Shown).

Quantification of the effects of optic nerve crush on dendritic complexity confirmed visual impressions. The 10 digitized cells from the retina of the crushed eye experienced, on average, a 49% decrease in complexity when compared to left eye controls (Fig 4C). The linear density of synapses in these retinas also fell. Cells from the crushed eye showed a 48% decrease in linear density on average when compared to right-eye controls (Control: 0.62 μm^-1^ v. Crushed-eye: 0.32 μm^-1^, Table 1) a more severe decrease than that seen in the DBA/2J experiment.

**Fig 7:**
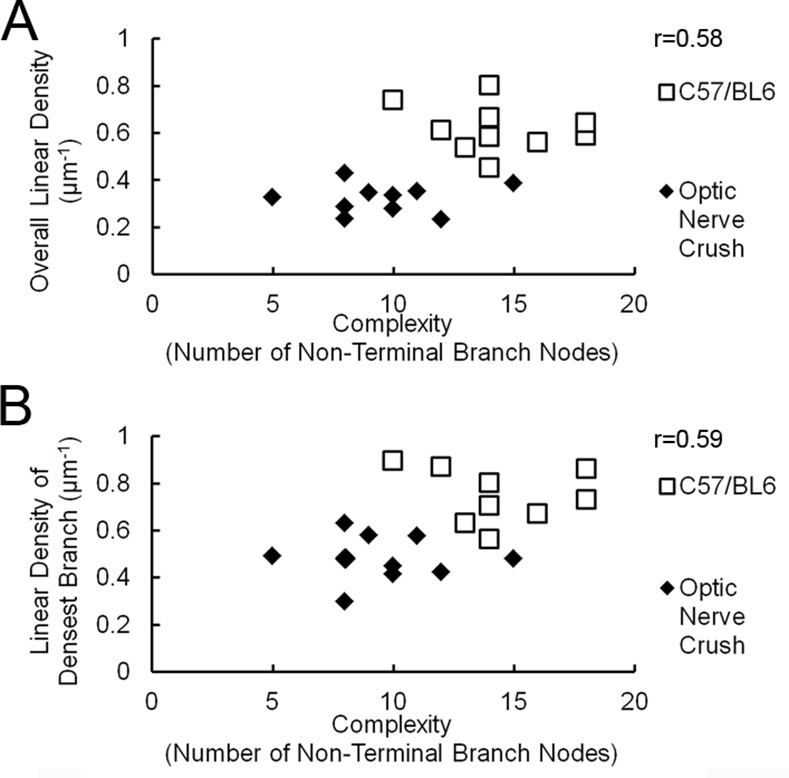
Correlation of synaptic density with complexity of the dendritic arbor in control C57bl/6 mice and in C57bl/6 mice after optic nerve crush. A, Scatterplot of synaptic density with complexity of the dendritic arbor in control retinas, and retinas after optic nerve crush. B, correlation of the synaptic density with complexity of the dendritic arbor for the densest branch.

The correlations between linear density and complexity of the dendritic arbor mirrored that seen in DBA/2J cells, albeit on a more drastic scale (Fig 7). On average, cells from the crushed eye were less complex and possessed lower overall linear densities than their control counterparts. When compared to the DBA/2J experiment, in fact, there was less overlap in the scatterplot between the control and affected cells, reflecting the more severe losses in dendritic complexity and linear density in the crush experiment (Figs 7A and 5A). As before, we ensured that these losses were not simply the result of primary branches comprising a greater portion of the total dendritic arbor by examining the relationship between complexity values and the linear density of the densest branches. Scatterplots representing this data confirmed that affected cells, when compared to cells from control retinas, showed both lower complexity values and linear densities for the densest branches (Fig 7B and 7C). Taken together, these results suggest that cells from the crushed eyes display, on a more drastic scale, the losses in dendritic complexity and linear density of excitatory input seen in cells from DBA/2J retinas when compared to D2-*Gpnmb* ^*+*^.

## Discussion

Synapse loss is observed in neurodegenerative diseases and a complement-driven pruning of synapses on ganglion cells has been proposed in the pathogenesis of glaucoma [8, 12]. In this scenario, components of the complement cascade would be aberrantly expressed in glaucomatous retinas and provide the signal for microglial cells to strip the synapses from the dendrites of ganglion cells. Alternatively, cell-autonomous processes within the ganglion cell itself may lead to synapse loss [34]. Synapse loss would in turn lead to a retraction and remodeling of the dendrites. If a major synaptic reduction occurred in glaucoma prior to significant dendrite atrophy, there should be a stage where ganglion cells show dendrites that are largely devoid of synaptic sites—these dendrites would be the candidates for remodeling.

Our method relies on the overexpression of fluorescently tagged PSD-95. Though this technique has been used in cell culture, tissue culture, and living animals before, there is concern whether the PSD-95-GFP puncta do indeed indicate synapses. In an earlier study, we demonstrated that in retinal ganglion cells transfected with PSD-95-GFP the puncta are colocalized with AMPA receptors and presynaptic elements labeled with antibodies against synaptic ribbon components [28]. Furthermore, the density of synaptic puncta in our sample was comparable to the density of synapses determined by electron microscopic reconstruction of a ganglion cell [35], and by immunohistochemical localization of synapses on the dendrites of microinjected ganglion cells [36, 37]. This indicates that overexpression of PSD-95-GFP does not necessarily lead to mislocalization of the protein. Our earlier study was performed in rabbit retinas and the density of puncta on non-glaucomatous mouse ganglion cells is somewhat higher, a finding that has also been reported by others [27, 38]. We furthermore use the SMI32 antibody to label the dendritic arbors of the ganglion cells we analyzed. This antibody detects the nonphosphorylated form of neurofilament H, and has been used to label alpha-like ganglion cells in a variety of species, including mice [3, 39, 40]. In mice, it preferentially labels ganglion cells with a large cell body and smooth dendrites that arborize in lamina 3 of the inner plexiform layer [40], however, it may label other types as well [41]. There are about 15 different types of ganglion cells in the mouse retina [41–45], and not all types may be susceptible to degeneration to the same degree [4, 46, 47]. However, the SMI32 positive cells in the glaucomatous retinas and in those after optic nerve crush show clear morphological signs of dendritic remodeling, and should therefore be suitable for our analysis.

In our sample of eyes with severe glaucoma, we did not observe dendrites that were denuded of synapses without being obviously remodeled. This is true both for the ganglion cells from glaucomatous retinas and for the cells after optic nerve crush. Our data thus suggest that there is no long period of time where dendrites persist though they have lost PSD-95 puncta. However, we cannot rule out that a reduction of synapse density or synaptic function occurs which does not lead to frank dendritic changes but which could still impact vision, as suggested by the recent report of early changes in multiarray recording from retinas after IOP increase [4]. Recently, it was also shown in the same publication that elevation of intraocular pressure with microbeads leads to a decrease in PSD-95 puncta on retinal ganglion cells even if there was no overt remodeling of the dendritic arbor [4]. This short-term manipulation may allow visualization of the rapid sequence between synapse pruning and dendritic decay.

It should also be remarked in this context that the presence of PSD-95 does not necessarily indicate a functional synapse. PSD-95, as the major scaffolding protein of the postsynaptic density, interacts with many other components of the synapse [21]. Amongst those are several subtypes of NMDR-type glutamate receptors [48], stargazin and related transmembrane AMPA receptor regulatory proteins (TARPs) that are involved in the surface expression of AMPA receptors [49–52], potassium channels [53], and others. PSD-95 is several times more abundant in the postsynaptic density than these [54]. Though PSD-95 undergoes dynamic turnover, it seems to be doing so at a slower rate than other synaptic proteins [14]. Therefore, impairment of synaptic function could precede the overt loss of PSD-95 puncta or dendritic remodeling, which may contribute to the early changes in multiarray recordings [4].

In DBA/2J glaucoma, the ganglion cells do not degenerate in synchrony [3, 55] and so we may have expected to see some cells undergoing early remodeling even in retinas with severe glaucoma. Although relatively few ganglion cells were quantitatively analyzed, many dozens more were inspected visually and there was never any evidence of a ganglion cell that had been stripped of synapses without remodeling. In addition, after optic nerve crush, which leads to dendrite remodeling and synapse loss on a faster time scale [56], the synchrony of the degeneration should make the intermediate, synapse-poor, stage easier to identify but no such cells were observed then either.

A possible interpretation would be that the loss of synapses is followed very quickly (hours or days) by consequences, direct or indirect, for dendritic viability. It is also possible that the synapses are not pruned from the dendrites simultaneously, in which case loss of a few synapses could be accompanied by a small effect on dendritic viability, events which gradually add up to the final gross dendritic remodeling. In either of those cases, a temporal separation of synapse pruning from remodeling would not have been observable in these experiments. The most likely reconciliation of our experiments and the previous ones would be that synaptic pruning causes remodeling (or vice versa) and there is a very short temporal separation between pruning and dendritic remodeling.
